# Multiomics Analysis Reveals Role of ncRNA in Hypoxia of Mouse Brain Microvascular Endothelial Cells

**DOI:** 10.3390/ijms26125629

**Published:** 2025-06-12

**Authors:** Qixin Shi, Shuai Zhang, Shaohua Li, Bin Zhang, Jin Xu, Yun-Gang Bai, Man-Jiang Xie, Jin Ma

**Affiliations:** Department of Aerospace Physiology, Air Force Medical University, Xi’an 710032, China; shiqixin41@fmmu.edu.cn (Q.S.); w327085122@126.com (S.Z.); soulcard@163.com (S.L.); fmmuzhangbin@yeah.net (B.Z.); 15980110320@163.com (J.X.); baiyun_1123@163.com (Y.-G.B.)

**Keywords:** ncRNA, hypoxia, oxidative stress, blood–brain barrier, multiomics, brain microvascular endothelial cells

## Abstract

Hypoxia leads to endothelial dysfunction and increased blood–brain barrier (BBB) permeability, promoting the incidence of diseases such as stroke and acute high-altitude illness. Brain microvascular endothelial cells (BMECs) are important structural and functional components of the BBB; however, the molecular changes that occur in BMECs during hypoxia remain unknown. We reported the molecular and functional changes in BMECs under hypoxia through whole-transcriptome sequencing, small RNA microarray, TMT quantitative proteomic, and untargeted metabolomic analyses. We found that hypoxia affected pathways such as ncRNA processing, the HIF-1 signaling pathway, the cell cycle, DNA replication, glucose metabolism, protein synthesis, and inflammation pathways. ncRNA processing was significantly downregulated. However, the levels of some miRNAs, tRNAs, tsRNAs, snoRNAs, lncRNAs, and circRNAs were significantly upregulated under hypoxia. These results suggest that ncRNAs may play an important role in oxidative stress and cellular adaptation to hypoxia, helping us understand the pathological process of BBB injury and providing potential targets for the treatment of BBB-related cerebrovascular diseases.

## 1. Introduction

The blood–brain barrier (BBB), which is formed by brain endothelial cells (ECs) interacting with mural cells, immune cells, glial cells, and neural cells in the neurovascular unit, is a highly selective microvascular structure that separates blood circulation and the central nervous system, and that plays a crucial role in maintaining normal brain function and central nervous system homeostasis [1]. Brain microvascular endothelial cells (BMECs) are important structural and functional components of the BBB. Tight junctions and transporters of BMECs strictly limit the movement of ions and molecules [2].

O_2_, as an electron acceptor and substrate, participates in multiple cellular processes. Oxygen homeostasis is crucial for cellular function. Hypoxia can alter cellular gene expression profiles and trigger multiple damage and adaptation mechanisms [3,4,5,6]. An amount of 1% O_2_ 24 h hypoxia was reported induce a decrease in endothelial cell transmembrane resistance and an increase in dextran transmembrane transport in mouse brain microvascular endothelial bEnd.3 cells [7]. In addition, CoCl_2_-induced hypoxia reduces transendothelial electrical resistance and increases FITC-dextran infiltration [8]. Multiple studies have demonstrated that oxygen and glucose deprivation (OGD) induce brain endothelial cell leakage [9,10,11]. These findings indicate that hypoxia compromises BBB permeability. An increase in BBB permeability can promote central nervous system damage. However, the underlying mechanism of hypoxia-induced endothelial dysfunction and BBB injury has yet to be clearly elucidated; the current studies focus on tight junctions, oxidative stress, and inflammatory response [9,10,12,13]. Understanding the changes in BMECs under hypoxia will help us better comprehend the pathological processes associated with BBB injury, thereby improving the treatment of stroke, acute high-altitude illnesses, and other diseases.

In recent years, rapid advances in microarray and high-throughput sequencing have provided powerful new tools for life science research. Genomics and transcriptomics have been widely applied in basic research, clinical diagnostics, and drug development [14]. Inspired by these approaches, researchers have also gradually developed proteomics and metabolomics techniques. mRNA is considered the “bridge” between genes and proteins, with proteins being the molecules that ultimately perform biological functions. The functional changes triggered by upstream macromolecules are eventually reflected at the metabolic level. Although transcriptomics, proteomics, and metabolomics each have unique advantages and disadvantages, their integration enables a holistic portrayal of the molecular landscapes in cells or tissues under specific conditions [15,16]. To date, multiomics technologies have been employed to study changes in BMECs in various diseases [17,18,19,20,21,22]. However, the understanding of the molecular changes in BMECs under hypoxia remains inadequate.

Transcriptomics refers to all the transcriptional products in an organism’s tissues or cells under specific conditions, including mRNA and noncoding RNA (ncRNA). ncRNA is transcribed from the genome but does not encode proteins. Based on their length, ncRNA can be roughly divided into long noncoding RNA (lncRNA, >200 nt) and small noncoding RNA (sncRNA, <200 nt). SncRNAs include microRNA (miRNA), small nucleolar RNA (snoRNA), transfer RNA (tRNA), tRNA-derived small RNA (tsRNA), small nuclear RNA (snRNA), and PIWI-interacting RNA (piRNA), among others. The broader category of lncRNAs also includes circular RNA (circRNA). ncRNAs play significant roles in gene expression at multiple levels. However, the change of ncRNAs in BMECs under hypoxia remains unclear.

To decipher the molecular and functional changes that occur in BMECs under hypoxia, we conducted whole-transcriptome sequencing, small RNA microarray, TMT quantitative proteomic, and untargeted metabolomic analyses on bEnd.3 cells after 24 h of hypoxia. Subsequently, bioinformatic analysis was used to investigate the molecular and metabolic mechanisms involved. Our findings revealed that hypoxia can affect pathways such as ncRNA processing, the HIF-1 signaling pathway, cell proliferation, and energy metabolism. Hypoxia significantly changes the levels of miRNAs, tRNAs, tsRNAs, snoRNAs, lncRNAs, and circRNAs in BMECs. Moreover, ncRNAs may play an important role in oxidative stress and cellular adaptation to hypoxia.

## 2. Results

### 2.1. Hypoxia Impairs BMEC Mitochondrial Function and Increases ROS and Apoptosis Levels

**Figure 1:**
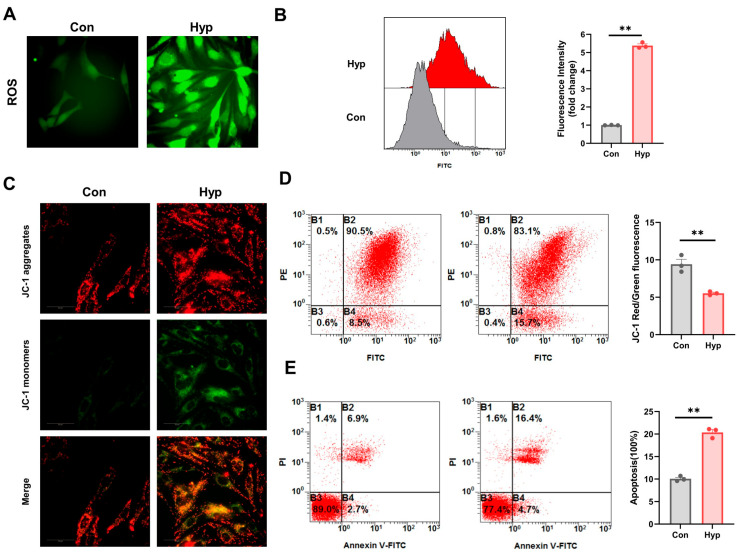
Hypoxia impairs BMEC mitochondrial function and increases ROS and apoptosis levels. (**A**,**B**) ROS level of BMECs under 24 h of hypoxia, scale bar = 50 μm. (**C**,**D**) MMP level of BMECs under 24 h of hypoxia, scale bar = 50 μm. (**E**) Apoptosis level of BMECs under 24 h of hypoxia. *n* = 3, ** *p* < 0.01 vs. Con; Student’s *t* test.

We used the fluorescent probe DCFH-DA to measure reactive oxygen species (ROS) levels in bEnd.3 cells after 24 h of 1% O_2_ hypoxia. We observed that BMECs loaded with DCFH-DA after 24 h of hypoxia exhibited significantly higher fluorescence intensity compared to control cells (Figure 1A). Similarly, the results of flow cytometry showed that the mean fluorescence intensity of hypoxic cells was significantly higher than that of the control group (Figure 1B), indicating that hypoxia increases ROS levels in BMECs. The JC-1 fluorescent probe was used to detect the mitochondrial membrane potential (MMP). The results showed that after 24 h of hypoxia, the red-to-green fluorescence ratio was significantly reduced, indicating a marked decrease in MMP (Figure 1C,D). Additionally, the results showed that BMEC apoptosis significantly increased compared to 24 h of hypoxia (Figure 1E). These findings suggest that hypoxia impairs BMEC mitochondrial function and increases ROS and apoptosis levels.

### 2.2. Differentially Expressed Genes (DEGs) in bEnd.3 Cells Under Hypoxic Conditions

**Figure 2:**
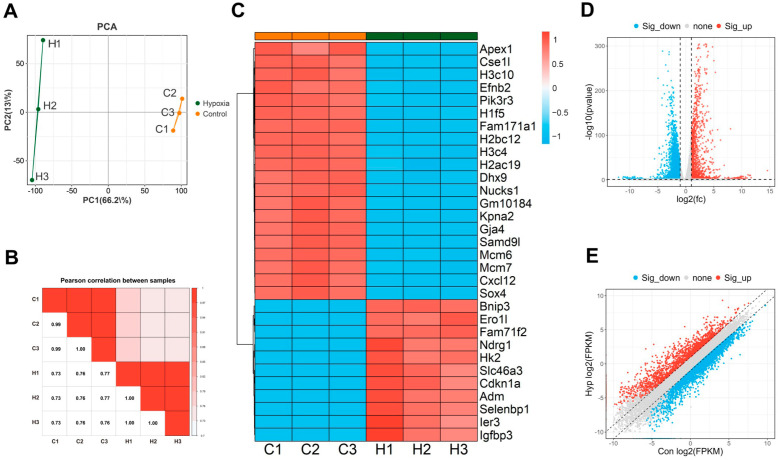
Hypoxia-induced DEGs in bEnd.3 cells. (**A**) A PCA plot. (**B**) Pearson correlation analysis plot. (**C**) A heatmap of the top 30 upregulated and downregulated DEGs between the control and hypoxia conditions. (**D**) A volcano plot of DEGs. (**E**) A scatter volcano plot of DEGs.

To comprehensively explore the impact of hypoxia on the molecular pathways in BMECs, we performed whole-transcriptome sequencing on bEnd.3 mouse brain microvascular endothelial cells after 24 h of hypoxia. The mRNA sequencing data were analyzed first. Principal component analysis (PCA) and Pearson correlation analysis revealed a high correlation between the three samples in the control and hypoxia groups (Figure 2A,B). The DEGs were visualized using heatmaps (Figure 2C), volcano plots (Figure 2D), and scatter volcano plots (Figure 2E). The results revealed that 1575 genes were significantly upregulated and 3261 genes were significantly downregulated (fold change (FC) ≥ 2, *p* < 0.05, Supplementary Table S1).

### 2.3. KEGG Analysis Reveals DEG Enrichment in DNA Replication, HIF-1 Signaling, and Glycolysis/Gluconeogenesis

To elucidate the biological functions of DEGs in different pathways, Kyoto Encyclopedia of Genes and Genomes (KEGG) and Gene Ontology (GO) enrichment analyses were performed.

**Figure 3:**
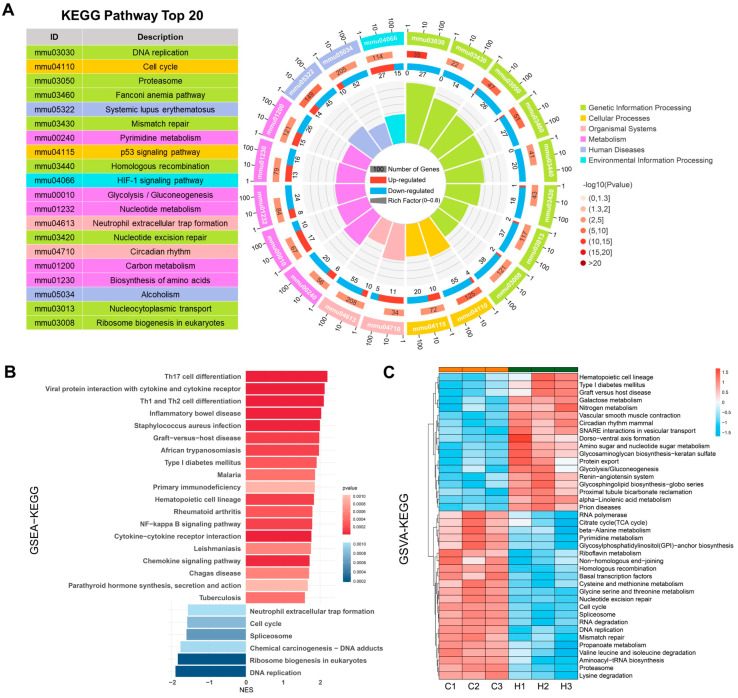
KEGG enrichment analysis. (**A**) A KEGG enrichment circle plot showing the top 20 pathways ranked by significance. From the outermost to innermost circles, the first circle represents pathway categories, with different colors indicating distinct categories; the second circle displays the total number of genes annotated to each pathway and the corresponding *p* value, where redder colors represent smaller *p* values; the third circle illustrates the ratio of upregulated to downregulated genes, with red denoting the proportion of upregulated genes and blue denoting the proportion of downregulated genes, with the numerical labels below indicating specific values; and the fourth circle shows the enrichment factor for each category, where each segment represents 0.1 and is calculated as the number of DEGs divided by the total number of genes in that pathway. (**B**) GSEA performed using the KEGG gene set. (**C**) A GSVA heatmap constructed using the KEGG gene set.

KEGG enrichment analysis revealed that the DEGs were significantly enriched in pathways such as DNA replication, the cell cycle, the proteasome, the Fanconi anemia pathway, mismatch repair, the p53 signaling pathway, homologous recombination, the HIF-1 signaling pathway, glycolysis/gluconeogenesis, nucleotide excision repair, and circadian rhythm (Figure 3A). Conventional enrichment analysis focuses only on DEGs filtered by thresholds, disregarding trends and the magnitude of changes in DEGs and other (non-differentially expressed) genes. Therefore, we performed gene set enrichment analysis (GSEA) using the KEGG gene set. The results revealed that inflammation-related pathways, such as the NF-κB signaling pathway, the chemokine signaling pathway, and cytokine-cytokine receptor interaction, were significantly upregulated, whereas the DNA replication, cell cycle, and spliceosome pathways were significantly downregulated (Figure 3B). Gene set variation analysis (GSVA) using the KEGG gene set revealed that pathways such as glycolysis/gluconeogenesis, circadian rhythm, and the renin-angiotensin system were significantly upregulated under hypoxia. Moreover, pathways related to DNA replication, the cell cycle, the TCA cycle, the proteasome, the spliceosome, mismatch repair, and homologous recombination were significantly downregulated (Figure 3C). These results indicate that hypoxia can promote the HIF-1 signaling pathway, glycolysis/gluconeogenesis, and inflammation, and can inhibit cell proliferation, cell cycle progression, DNA damage repair, protein synthesis, and energy metabolism.

### 2.4. GO Analysis Reveals DEGs Enrichment in ncRNA Processing

**Figure 4:**
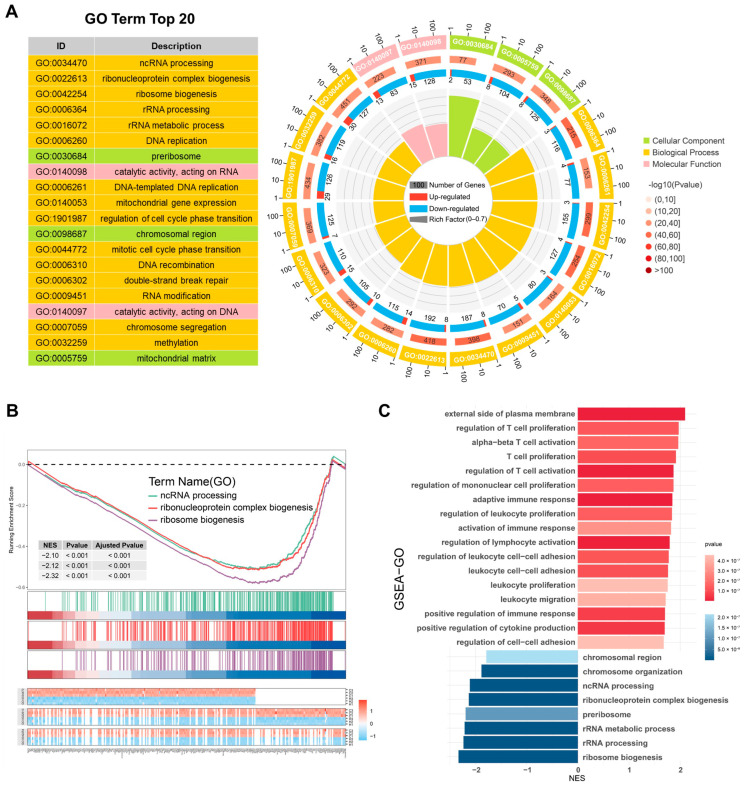
GO enrichment analysis DEGs enrichment in ncRNA processing. (**A**) A GO enrichment circle plot. (**B**) GSEA curves of DEGs for the hypoxia vs. control comparison. (**C**) GSEA using the GO gene set.

GO analysis includes three categories: the biological process (BP), the cellular component (CC), and the molecular function (MF). The DEGs were enriched in the BP terms ncRNA processing, ribonucleoprotein complex biogenesis, ribosome biogenesis, rRNA processing, DNA replication, mitochondrial gene expression, RNA modification, and methylation; the CC terms preribosome, the chromosomal region and the mitochondrial matrix; and the MF term catalytic activity acting on RNA and DNA (Figure 4A). GSEA of the GO gene set revealed that the significantly enriched pathways included those related to the activation of the immune response, the positive regulation of cytokine production, and cell–cell adhesion regulation. Moreover, processes such as ncRNA processing, rRNA processing, ribonucleoprotein complex biogenesis, and ribosome biogenesis were significantly downregulated (Figure 4B,C). These results indicate that hypoxia may regulate inflammation and biosynthesis, and energy metabolism through ncRNA.

### 2.5. Hypoxia Alters Small Noncoding RNA Expression in BMECs

GO enrichment analysis indicated that hypoxia significantly affected ncRNA processing in BMECs. To investigate potential changes in ncRNAs, we applied whole-transcriptome sequencing and small RNA microarrays to assess the levels of small noncoding RNAs.

**Figure 5:**
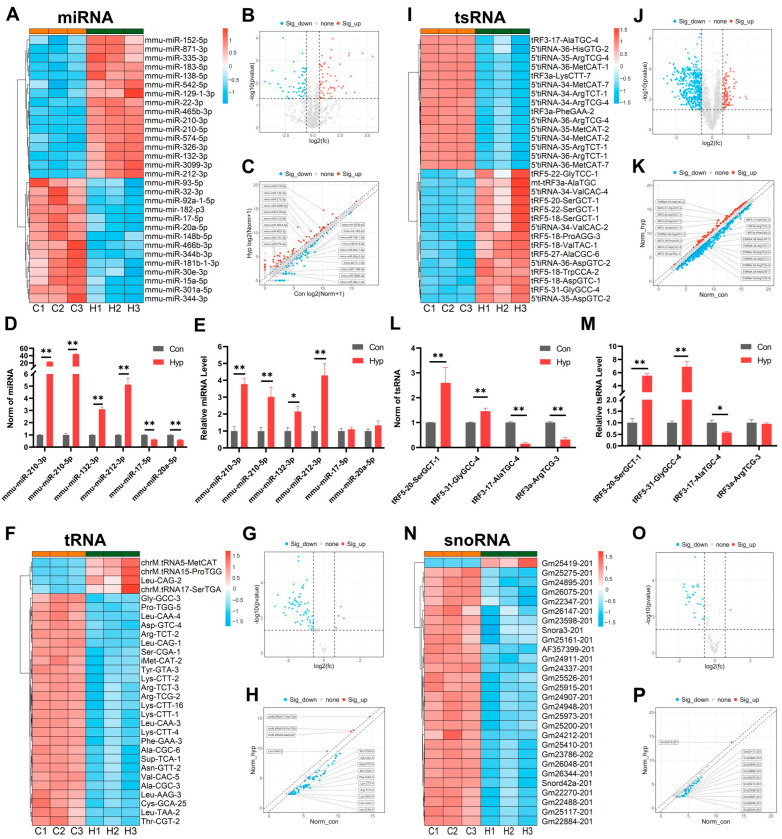
Hypoxia alters small noncoding RNA expression in BMECs. (**A**–**C**) A heatmap, volcano plot, and scatter plot of differentially expressed miRNAs. (**D**,**E**) The levels of 6 miRNAs in whole transcriptome sequencing data and RT–qPCR data (*n* = 3). (**F**–**H**) A heatmap, volcano plot, and scatter plot of differentially expressed tRNAs. (**I**–**K**) A heatmap, volcano plot, and scatter plot of differentially expressed tsRNAs. (**L**,**M**) The levels of the 4 tsRNAs in small RNA microarray data and RT–qPCR data (*n* = 3). (**N**–**P**) A heatmap, volcano plot, and scatter plot of differentially expressed snoRNAs. * *p* < 0.05, ** *p* < 0.01 vs. Con; Student’s *t* test.

MicroRNAs (miRNAs) are endogenous noncoding RNA molecules of approximately 18–22 nt that can play important regulatory roles by targeting the 3′ UTRs of target mRNAs to promote their cleavage or translational repression [23]. Whole-transcriptome sequencing identified 69 significantly upregulated and 64 significantly downregulated miRNAs (Figure 5A–C, FC ≥ 1.5, *p* < 0.05, Supplementary Table S2). We performed RT-qPCR on several of the miRNAs whose expression significantly differed. The results confirmed that miR-210-3p, miR-210-5p, miR-132-3p, and miR-212-3p were significantly upregulated under hypoxia (Figure 5D,E). Our other study showed that miR-212-3p inhibited cell cycle progression and cell proliferation by regulating MCM2 in BMECs under hypoxia [24]. In addition, previous studies and our unpublished data indicated that hypoxia induced mitochondrial damage and apoptosis through miR-210-3p [25]. These results suggest that miR-212-3p and miR-210-3p play important roles in oxidative stress and cellular adaptation to hypoxia.

Since their discovery more than 50 years ago, tRNAs have received widespread attention from researchers. Recent studies have shown that the expression patterns of tRNA-coding genes exhibit tissue and cell specificity. The expression and function of tRNAs are dynamically regulated by posttranscriptional RNA modifications, and alterations in tRNA abundance or functionality may affect cell functions and contribute to various diseases [26]. Small RNA microarrays revealed that tRNA^Leu(CAG)^, mt-tRNA^Met(CAT)^, mt-tRNA^Pro(TGG)^, and mt-tRNA^Ser(TGA)^ were significantly upregulated, whereas 57 tRNAs were significantly downregulated (Figure 5F,G; FC ≥ 1.5, *p* < 0.05, Supplementary Table S3). These findings indicate that tRNA levels, including mitochondrial tRNA levels, undergo significant changes under hypoxia.

Mature tRNAs or precursor tRNAs can be specifically cleaved into tsRNAs. tsRNAs include tRNA-derived fragments (tRFs) and tRNA halves (tiRNAs). tsRNAs perform various biological activities, including AGO-dependent and AGO-independent translational repression, binding proteins to affect mRNA stability, and acting as paternal epigenetic factors to alter offspring gene transcription. Additionally, tiRNAs can be induced under stress conditions such as amino acid starvation, hypoxia, oxidative stress, and viral infection [27]. Small RNA microarrays indicated that 97 tsRNAs were significantly upregulated, whereas 407 tsRNAs were significantly downregulated, under hypoxia (Figure 5I–K, FC ≥ 1.5, *p* < 0.05, Supplementary Table S4). RT-qPCR revealed significant changes in tRF5-20-SerGCT-1, tRF5-31-GlyGCC-4, and tRF3-17-AlaTGC-4 (Figure 5L,M). These findings suggest that hypoxia affects tsRNA levels in BMECs.

Small nucleolar RNAs (snoRNAs) were first discovered in mammalian cells in 1960. These proteins are primarily involved in posttranscriptional modifications, forming small nucleolar ribonucleoproteins (snoRNPs) with associated proteins. snoRNPs guide 2′-O-ribose methylation and pseudouridylation of rRNAs and snRNAs, enhancing ligand interactions and facilitating 3D folding to fine-tune ribosome and spliceosome functions [28]. Our small RNA microarray identified one significantly upregulated and 27 significantly downregulated snoRNAs in the hypoxia group (Figure 5N–P, FC ≥ 1.5, *p* < 0.05, Supplementary Table S5).

### 2.6. CircRNAs and lncRNAs Regulate Gene Expression Through ceRNA Mechanisms During Hypoxia

**Figure 6:**
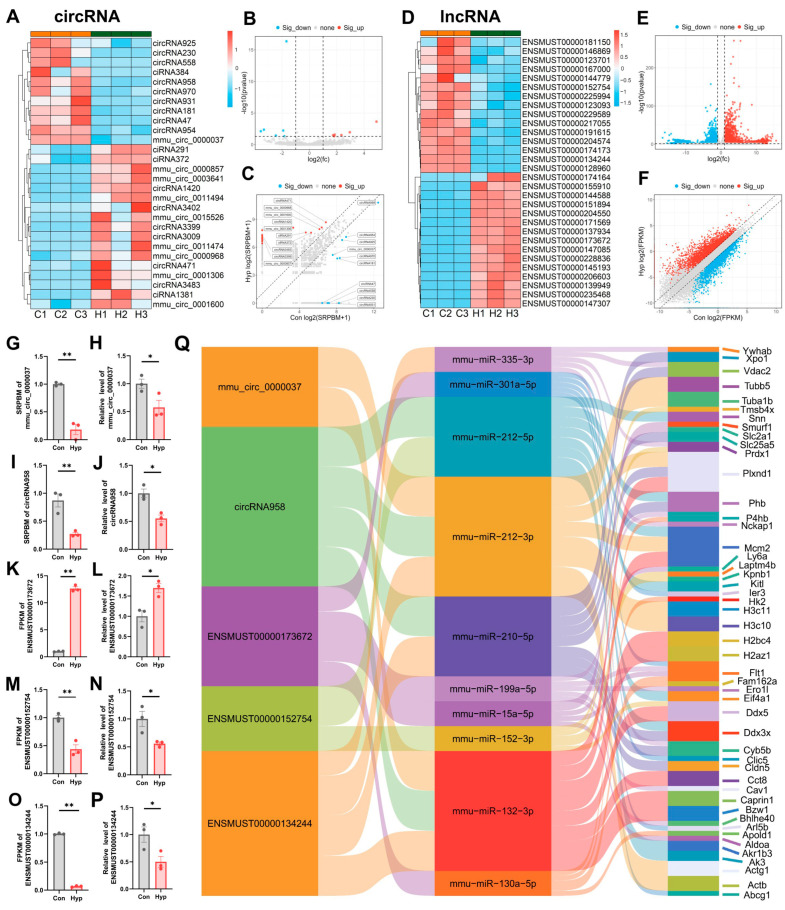
CircRNAs and lncRNAs act as ceRNAs regulate gene expression during hypoxia. (**A**,**D**) Heatmap of differentially expressed circRNAs and lncRNAs. (**B**,**E**) Volcano plot. (**C**,**F**) Scatter volcano plot. (**G**–**P**) Levels of circRNAs and lncRNAs from whole-transcriptome sequencing data and RT-qPCR data (*n* = 3). (**Q**) Sankey diagram of ceRNAs (*n* = 3). * *p* < 0.05, ** *p* < 0.01 vs. Con; Student’s *t* test.

circRNAs are single-stranded noncoding RNA molecules lacking 5′ caps and 3′ poly(A) tails that form covalently closed, circular structures. Most circRNAs arise from the backsplicing of precursor mRNAs. circRNAs regulate transcription, splicing, mRNA stability, translation, and signaling pathways by forming R-loops with their production sites, interacting with proteins to form complexes, acting as miRNA sponges, or binding directly to mRNAs. In addition to their noncoding functions, recent studies have demonstrated that some circRNAs can be translated [29]. Whole-transcriptome sequencing revealed that 17 circRNAs were significantly upregulated and 11 circRNAs were significantly downregulated in bEnd.3 cells after 24 h of hypoxia (Figure 6A–C, FC ≥ 2, *p* < 0.05, Supplementary Table S6). We performed RT–qPCR for selected circRNAs with significant differences. The results revealed that circRNA958 and mmu_circ_0000037 were significantly downregulated under hypoxia (Figure 6G–J).

Linear noncoding transcripts longer than 200 nt are defined as lncRNAs, which are less conserved across species than protein-coding mRNAs but exhibit greater cell specificity. lncRNAs participate in various cellular processes, such as proliferation, differentiation, energy metabolism, and signal transduction, by acting as miRNA sponges, interacting with chromatin-modifying proteins, regulating enhancer activity, or contributing to biomolecular condensates or phase-separated regions [30]. Whole-transcriptome sequencing identified 2969 significantly upregulated and 2400 significantly downregulated lncRNAs (Figure 6D–F, FC ≥ 2, *p* < 0.05, Supplementary Table S7). RT–qPCR revealed significant changes in the expression of ENSMUST00000173672, ENSMUST00000134244, and ENSMUST00000152754 (Figure 6K–P).

Competing endogenous RNAs (ceRNAs) have attracted considerable attention in recent years. Unlike “ncRNA”, “ceRNA” refers to a regulatory model rather than a specific type of RNA. For example, miRNAs can inhibit the translation of mRNAs by binding to them, while some RNAs act as miRNA sponges by competitively binding miRNAs to regulate gene expression. The most common ceRNAs are lncRNAs and circRNAs. High-throughput sequencing and bioinformatics analyses allow for the construction of ceRNA networks, providing a more comprehensive understanding of cellular regulatory networks and deeper insights into gene functions and molecular mechanisms. On the basis of the RT-qPCR results, we constructed a ceRNA network and visualized some of the results in a Sankey diagram (Figure 6Q).

### 2.7. Proteomic GSEA Reveals ncRNA Processing Was Significantly Downregulated

**Figure 7:**
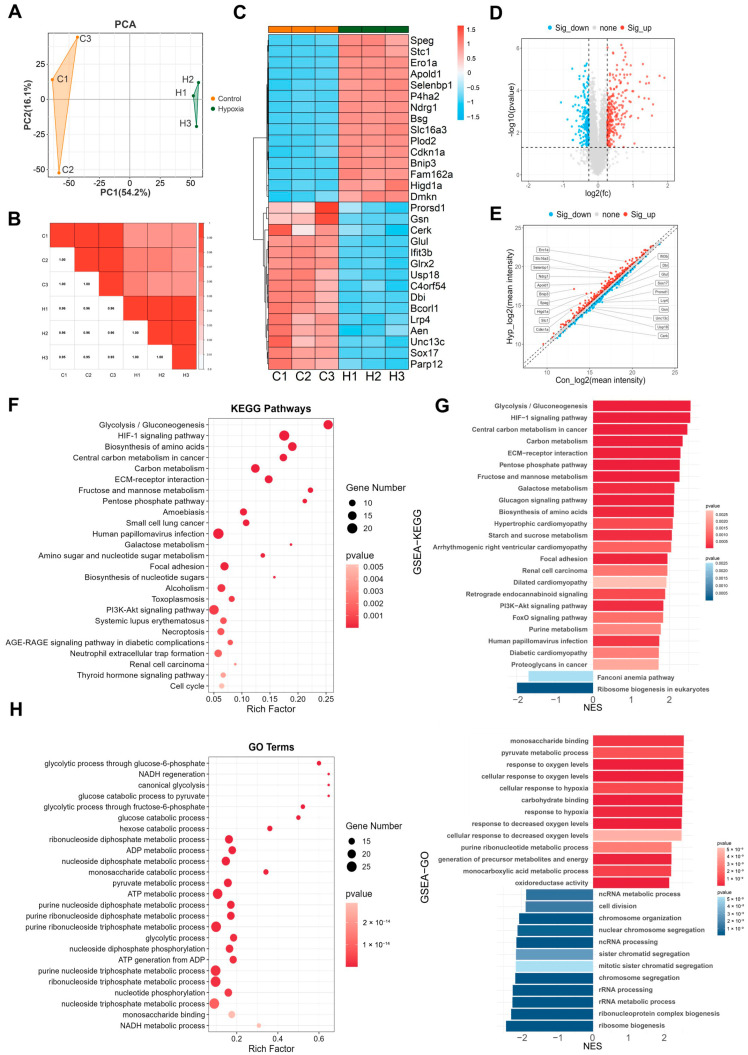
Proteomic GSEA reveals that ncRNA processing was significantly downregulated. (**A**) A PCA plot. (**B**) A Pearson correlation analysis plot. (**C**) A heatmap of the top 30 differentially expressed proteins. (**D**) A volcano plot. (**E**) A scatter volcano plot. (**F**) KEGG enrichment analysis. (**G**) GSEA performed using the KEGG gene set. (**H**) GO enrichment analysis. (**I**) GSEA performed using the GO gene set.

The genetic central dogma states that genetic information flows from genes to proteins via mRNA; however, mRNA levels do not always correspond to protein expression due to translation regulation, posttranslational modifications, and structural changes. Therefore, proteomic analysis is essential for further investigating the effects of hypoxia on BMECs. We performed TMT-based proteomic quantification on bEnd.3 cells exposed to 24 h of hypoxia. PCA and Pearson correlation analysis revealed a high correlation between the three samples in the control and hypoxia groups (Figure 7A,B). The differentially expressed proteins were visualized using heatmaps (Figure 7C), volcano plots (Figure 7D), and scatter volcano plots (Figure 7E). The results revealed that 248 proteins were significantly upregulated, whereas 201 proteins were significantly downregulated (FC ≥ 1.2, *p* < 0.05, Supplementary Table S8).

The KEGG enrichment analysis revealed that the differentially expressed proteins were involved in pathways such as glycolysis/gluconeogenesis, the HIF-1 signaling pathway, amino acid biosynthesis, carbon metabolism, and the cell cycle (Figure 7F). GSEA was performed using the KEGG gene set, and the results revealed the significant upregulation of genes related to glycolysis/gluconeogenesis, the HIF-1 signaling pathway, carbon metabolism, amino acid biosynthesis, the PI3K/Akt signaling pathway, etc. (Figure 7G). GO enrichment analysis revealed that the differentially expressed proteins were involved in pathways such as the glycolytic process of glucose-6-phosphate, NADH regeneration, glucose metabolism to pyruvate, ADP metabolism, pyruvate metabolism, and ATP metabolism (Figure 7H). GSEA was conducted using the GO gene set, and the results revealed that pyruvate metabolism and the response to oxygen were significantly upregulated, whereas ncRNA processing, cell division, chromosome separation, and ribosome biogenesis were significantly downregulated (Figure 7I). The changes in ncRNA processing in proteomics further support our conclusion.

### 2.8. Protein-Protein Interaction Analysis Identifies Hub Genes Involved in Glycolytic Processes

**Figure 8:**
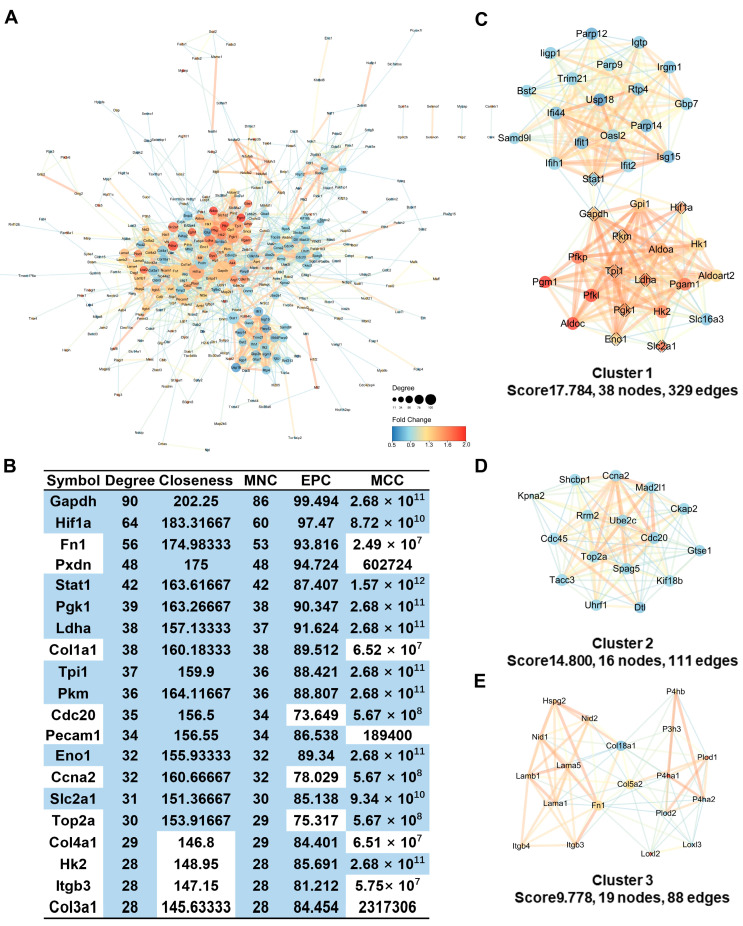
Protein–protein interaction analysis indicates that hub genes are involved in glycolytic processes. (**A**) A PPI network constructed with STRING and visualized using Cytoscape. Larger nodes represent proteins with more interactions. The node colors represent fold changes, and darker colors indicate greater changes, with red indicating significantly upregulated proteins and blue indicating significantly downregulated proteins. The thickness and color of the connecting lines represent the combined score, which indicates the support of the data. The thicker the line and the redder the color, the stronger the interaction between the two proteins. The threshold is 0.4. (**B**) Hub genes defined by the CytoHubba plugin in Cytoscape. (**C**–**E**) The top three gene clusters selected by the MCODE plugin in Cytoscape have the highest scores, among which the hub genes highlighted by diamond borders have the highest scores.

The STRING database https://string-db.org (accessed on 18 November 2023) systematically compiles and integrates protein-protein interaction (PPI) data, including both physical and functional relationships [31]. To further investigate the changes in complex molecular networks in BMECs under hypoxia, we constructed a PPI network, which comprised 442 nodes and 1769 edges (interaction score > 0.4), via the STRING database and then visualized and analyzed the results via Cytoscape (Figure 8A). The cytoHubba (version 0.1) plugin in Cytoscape ranks PPI network nodes on the basis of network characteristics [32]. We used cytoHubba to identify hub genes by selecting the top 20 genes from four algorithms (degree, closeness, MNC, and EPC) and the top 40 genes from the MCC algorithm (Figure 8B). The nine identified hub genes were glyceraldehyde-3-phosphate dehydrogenase (GAPDH), HIF1A, signal transducer and activator of transcription 1 (STAT1), phosphoglycerate kinase 1 (PGK1), lactate dehydrogenase A (LDHA), triose-phosphate isomerase 1 (TPI1), pyruvate kinase M1/2 (PKM), enolase 1 (ENO1), and solute carrier family 2 member 1 (SLC2A1). HIF1A and STAT1 are important transcription factors during hypoxia. GAPDH, PGK1, LDHA, TPI1, PKM, and ENO1 are associated with glycolysis. SLC2A1 is an important glucose transporter in the blood-brain barrier that plays a crucial role in cellular glucose metabolism. The overexpression of SLC2A1 can promote glycolysis and cell proliferation.

The highly interconnected regions of PPI networks may play a greater role in diseases. The MCODE plugin of Cytoscape can search for highly interconnected regions in PPI networks, which helped us to focus on key subnetworks and genes. Therefore, we used the MCODE plugin to select 13 subnetworks from the PPI network and visualized the top three gene clusters with the highest scores (Figure 8C–E). The highest scoring subnetwork had a score of 17.784, with a total of 38 nodes and 329 edges. This subnetwork included nine hub genes selected by the cytoHubba plugin (Figure 8C).

### 2.9. Integrative Transcriptomic and Proteomic Analysis

**Figure 9:**
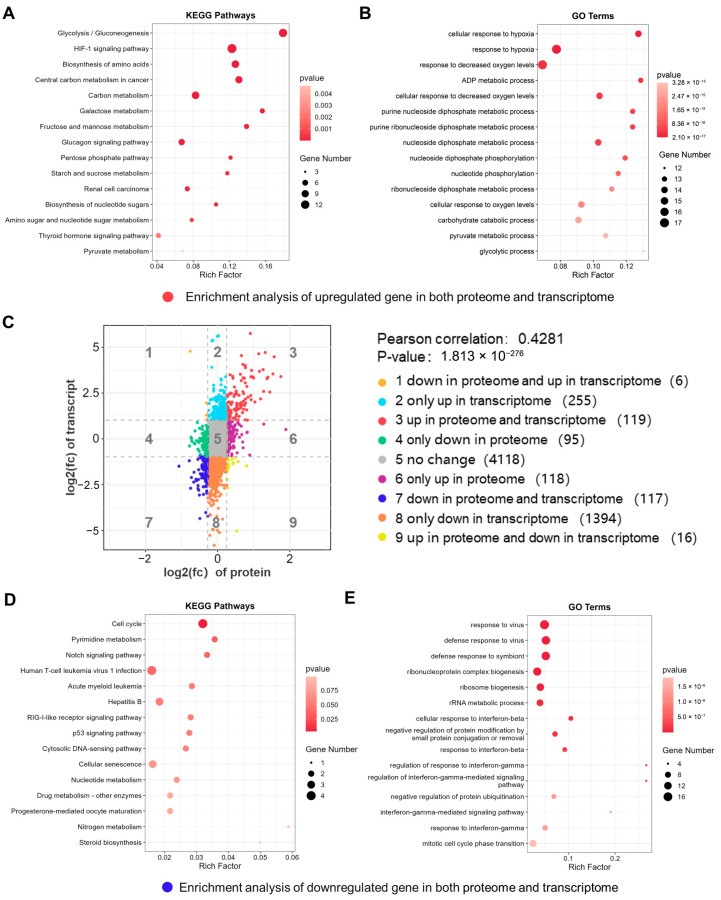
Integrated transcriptomic and proteomic analysis. (**A**,**B**) KEGG and GO enrichment analyses of genes upregulated at both the transcriptomic and proteomic levels. (**C**) A nine-quadrant plot of the integrated transcriptomic and proteomic analysis, with a transcriptomic threshold FC ≥ 2 and a proteomic threshold FC ≥ 1.2. (**D**,**E**) KEGG and GO enrichment analyses of genes downregulated at both the transcriptomic and proteomic levels.

To explore the effects of hypoxia on BMEC gene expression more precisely, we conducted an integrated analysis of the transcriptomic and proteomic data and visualized the results in a nine-quadrant plot (Figure 9C), applying thresholds of FC ≥ 2 for the transcriptomic and FC ≥ 1.2 for the proteomic. It should be noted that the nine-quadrant plot reflects the relationships between fold changes across different omics datasets without incorporating *p* values. Among the 6238 genes matched between the proteomic and transcriptomic, 22 (0.35%) presented opposing expression patterns between proteins and corresponding mRNAs (quadrants 1 and 9), 1649 (26.43%) presented changes in mRNA levels without changes in protein expression (quadrants 2 and 8), and 213 (3.41%) mRNAs presented no changes, but the corresponding proteins presented changes (quadrants 4 and 6). These discrepancies may result from posttranscriptional, translational, or posttranslational regulation.

Furthermore, 119 genes were simultaneously upregulated at the mRNA and protein levels (quadrant 3), whereas 117 were downregulated at both levels (quadrant 7). Genes with consistent transcriptional and translational changes may play pivotal roles during hypoxia. We performed enrichment analysis of these two subsets of genes. Genes upregulated at both the transcriptomic and proteomic levels were significantly enriched in processes such as glycolysis/gluconeogenesis, the HIF-1 signaling pathway, amino acid biosynthesis, carbon metabolism, cellular responses to hypoxia, ADP metabolism, and nucleotide metabolism (Figure 9A,B). Genes downregulated in both the transcriptomic and proteomic levels were significantly enriched in pathways such as the cell cycle, Notch signaling, p53 signaling, and responses to viruses and interferons (Figure 9D,E).

### 2.10. Metabolomic Analysis Shows Hypoxia Alters Purine Metabolism and Pyrimidine Metabolism

**Figure 10:**
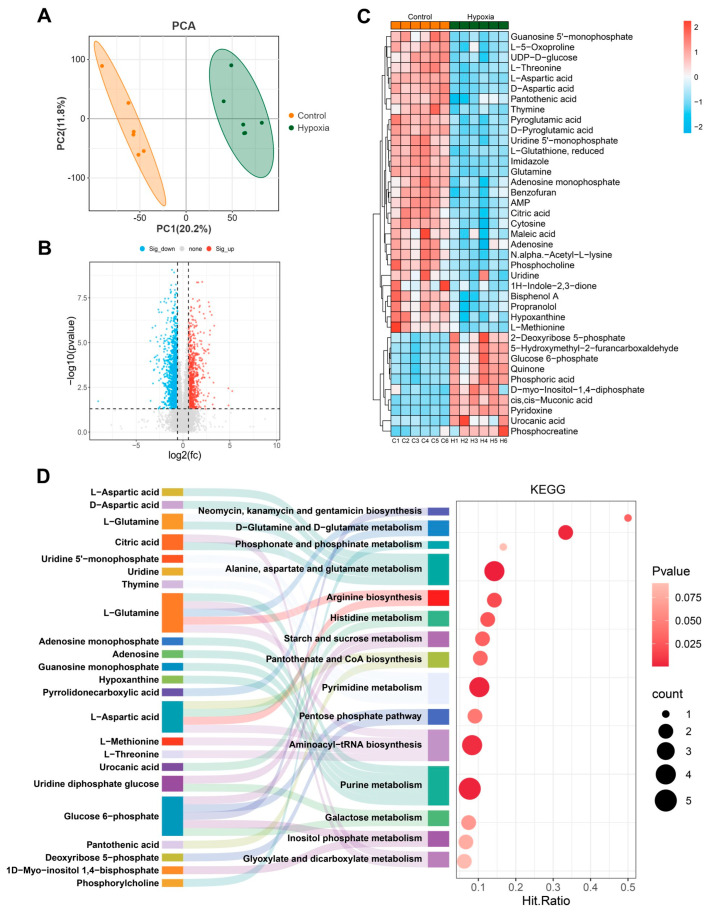
Metabolomic analysis shows that hypoxia alters purine metabolism and pyrimidine metabolism. (**A**) A PCA plot. (**B**) A volcano plot of primary metabolites, with thresholds of FC ≥ 1.5, *p* < 0.05, and VIP ≥ 1. (**C**) A heatmap of secondary metabolites. (**D**) KEGG enrichment analysis of the top 15 significant pathways. The x-axis shows the hit ratio, which is calculated as the number of differentially abundant metabolites in a pathway divided by the total background metabolites in that pathway. The bubble size represents the number of differentially abundant metabolites, while the bubble color indicates the *p* value, with darker colors signifying greater significance. The Sankey diagram on the left displays the differentially abundant metabolites associated with each pathway.

**Figure 11:**
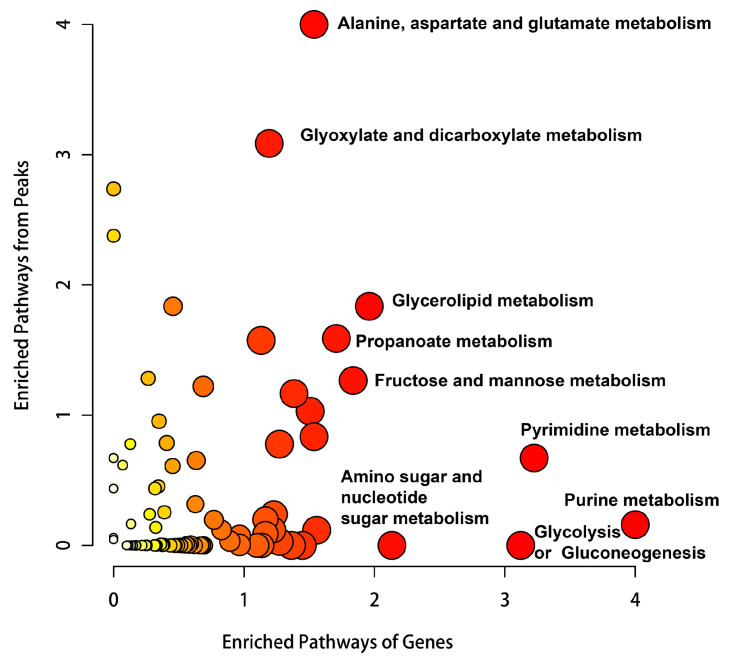
KEGG enrichment analysis combining transcriptomic and untargeted metabolomic data. The color represents the *p* value of the integrated analysis, where redder colors represent smaller *p* values.

Cellular functions are collectively governed by DNA, RNA, proteins, and metabolites. Changes in the levels of upstream macromolecules, such as nucleic acids and proteins, ultimately manifest in changes at the metabolic level. Therefore, we conducted untargeted metabolomic analysis on bEnd.3 cells after 24 h of hypoxia. The PCA results revealed a high correlation between the six samples in the control and hypoxia groups (Figure 10A). Differential analysis revealed that 841 primary metabolites were significantly upregulated and 1361 were significantly downregulated upon hypoxia (Figure 10B). Additionally, secondary metabolite identification revealed 19 significantly upregulated and 44 significantly downregulated secondary metabolites (Figure 10C, FC ≥ 1.5, *p* < 0.05, VIP ≥ 1, Supplementary Table S9). KEGG enrichment analysis revealed that the differentially abundant metabolites were significantly enriched in pathways such as purine metabolism, aminoacyl-tRNA biosynthesis, pyrimidine metabolism, and D-glutamine and D-glutamate metabolism (Figure 10D). Additionally, we performed KEGG enrichment analysis combining the transcriptomic and untargeted metabolomic data using MetaboAnalyst 6.0 https://www.metaboanalyst.ca (accessed on 12 March 2024). The results suggest significant enrichment in pathways such as purine metabolism, pyrimidine metabolism, and glycolysis/gluconeogenesis (Figure 11). These findings further indicate that hypoxia significantly affects DNA and RNA-related biological processes.

## 3. Discussion

Many studies have investigated the effects of hypoxia on gene expression profiles [33,34,35,36]. However, it is difficult to comprehensively describe the effects of hypoxia using a single omics approach. Multiomics can more accurately describe the overall molecular characteristics after hypoxia, which aids in understanding the impact of hypoxia on the functional status and signaling network of BMECs and better reveals the molecular mechanisms of stroke, AHI, and other diseases.

For this reason, we conducted transcriptomic, small RNA microarray, TMT quantitative proteomic, and untargeted metabolomic analyses on bEnd.3 cells after 24 h of hypoxia. Enrichment analysis indicated that hypoxia affects pathways such as the ncRNA processing, HIF-1 signaling, cell cycle, DNA replication, glucose metabolism, protein synthesis, and inflammation pathways. The cell cycle and DNA replication are significantly inhibited, whereas glycolysis/gluconeogenesis and the HIF-1 signaling pathway are significantly activated. These findings are in line with previous meta-analyses of hypoxic transcriptomics in human cells [37]. The meta-analysis comprising 430 RNA-seq samples from 43 individual studies, including 34 different human cell types, showed that cell cycle progression, DNA replication and repair, ribosome/rRNA biogenesis, mitochondrial respiratory electron transport, and complex I biogenesis were repressed by hypoxia, and HIF-related pathways and glucose metabolism were upregulated by hypoxia. These findings indicate that cellular adaptation to hypoxia may be highly conserved across different species.

Emerging studies have indicated that ncRNAs play important roles in inflammation, tumors, stroke, myocardial infarction, pulmonary arterial hypertension, and other diseases under hypoxia [38,39,40,41,42,43]. In the present study, transcriptomics and proteomics indicated that hypoxia significantly affects ncRNA processing. Metabolomic analysis showed that hypoxia alters purine metabolism and pyrimidine metabolism. These results reveal the impact of hypoxia on ncRNA processing at different levels. Furthermore, our study showed that hypoxia significantly changed the ncRNA levels, such as miRNAs, tRNAs, tsRNAs, snoRNAs, lncRNAs, and circRNAs. Interestingly, similar to previous studies, our results also indicated that ncRNA processing is downregulated during hypoxia [44]. However, some ncRNAs are significantly upregulated during hypoxia, such as miR-210-3p, miR-212-3p, tRNA^Leu(CAG)^, tRF5-20-SerGCT-1, and tRF5-31-GlyGCC-4, suggesting that these ncRNAs may play a more important role in hypoxia injury and adaptation. Our previous research confirmed that miR-212-3p inhibits cell cycle progression and cell proliferation by regulating MCM2 in BMECs under hypoxia [24].Many studies and our unpublished data indicated that hypoxia induced mitochondrial damage and apoptosis through miR-210-3p [25]. In addition, the previous study has demonstrated that hypoxia-mediated miR-212-3p downregulation enhances the progression of intrahepatic cholangiocarcinoma through the upregulation of Rab1a [45]. miR-210-3p-enriched extracellular vesicles from hypoxic neuroblastoma cells stimulate migration and the invasion of target cells [46]. miR-210 promotes cardiomyocyte survival and mediates exercise-induced cardiac protection against ischemia/reperfusion injury [47]. These studies have demonstrated that specific ncRNAs (e.g., miR-210-3p, miR-212-3p) play important roles in many diseases. Further experiments are needed to investigate the role of other ncRNAs in different diseases under hypoxia.

Additionally, we found that both the mRNA and protein expression levels of HIF1A were increased, indicating that in hypoxic cells, hypoxia not only inhibits the degradation of HIF1A through PHD but also promotes HIF1A gene expression, with both mechanisms working synergistically. However, previous studies have shown that hypoxia does not always upregulate the mRNA levels of HIF1A and sometimes inhibits its expression, which may be related to the type of cell, the severity of the hypoxia, and the experimental methods used for detection [33,37,48,49].

mRNAs are considered the “bridge” between genes and proteins, with proteins being the molecules that ultimately execute biological functions. Therefore, we conducted a comprehensive analysis of the proteomic and transcriptomic results. The results revealed that out of the 6238 gene–protein pairs, 119 were simultaneously upregulated at the mRNA and protein levels, whereas 117 were downregulated at both levels. Thus, a total of 236 genes (50.1%) changed synchronously at the transcriptional and translational levels, indicating that more than half of the differential protein changes may be related to mRNA changes. However, many protein expression changes are inconsistent or even opposite to those of the corresponding mRNAs, which may be caused by differences in RNA and protein stability, posttranscriptional modifications of RNA, or posttranslational processing of proteins. Moreover, the analysis revealed many genes whose mRNA levels changed but whose corresponding protein levels did not change (1649). This finding indicates that transcription levels are not sufficient to predict protein levels, and high-quality data that quantify gene expression at different levels are essential for a complete understanding of biological processes [50].

Ordinary enrichment analysis focuses only on the differentially expressed factors screened according to defined thresholds but does not consider the trend and degree of variation or changes in the abundance of other factors that do not meet the defined thresholds, which can result in the loss of a large amount of information in omics results. Therefore, we also conducted GSEA, which can utilize the differential fold information of all genes to detect gene sets that are not significantly differentially expressed but have consistent expression trends. It can also clearly display whether the pathway is upregulated or downregulated. It is important to note that GSEA results provide trends of upregulation or downregulation across gene sets, but the upregulation or downregulation of genes does not necessarily imply the activation or inhibition of the corresponding signaling pathways. For example, while the KEGG cell cycle signaling pathway was downregulated in our transcriptomic data, the levels of cyclin-dependent kinase inhibitor 1A (CDKN1A/p21), CDKN1B/p27, and CDKN1C/p57 were upregulated. This upregulation weakens the GSEA score for the pathway, but in fact, the increased levels of these inhibitors are consistent with the overall inhibition of the pathway. If enrichment analysis algorithms could consider and optimize this situation, they would more accurately describe the activation and inhibition of gene sets under specific conditions.

The limitations of the present study mainly lie in two aspects. (1) The present study identifies significant molecular changes. However, the functional experiments to validate the roles of specific ncRNAs are limited, and we will explore the mechanism in our future research. (2) This study only explored the molecular and functional changes in bEnd.3 cells and may not fully represent in vivo conditions, limiting the generalizability of findings to human BBB physiology. Furthermore, the results are not directly applicable to clinical applications for stroke or high-altitude illness treatment, and we will study this in the future.

## 4. Materials and Methods

### 4.1. Cell Culture and Treatment

The mouse brain microvascular endothelial bEnd.3 cell line was purchased from iCell (Shanghai, China). The cells were grown in specific complete culture media (cat. no. iCell-128-0001, iCell, Shanghai, China) supplemented with 10% fetal bovine serum (InCellGene, Burlington, ON, Canada) and 1% penicillin/streptomycin (InCellGene, Burlington, ON, Canada) at 37 °C in 5% CO_2_. The cells were seeded and cultured for 24 h. bEnd.3 cells were exposed to low oxygen gas (94% N_2_, 5% CO_2_, and 1% O_2_) for 24 h as the hypoxia group, and cells cultured under normoxic conditions were used as the control group. All experiments were repeated 3 times (*n* = 3).

### 4.2. Reactive Oxygen Species Assay

The cells were seeded and cultured for 24 h. Then, the cells were subjected to hypoxia exposure. The ROS level was analyzed with the Reactive Oxygen Species Assay Kit (Beyotime, Shanghai, China) according to the manufacturer’s instructions. The images were observed using an Operetta CLS high content analysis system (PerkinElmer, Wellesley, MA, USA). An EPICS XL flow cytometer (Beckman, Brea, CA, USA) was used for flow cytometry.

### 4.3. Mitochondrial Membrane Potential Assay

The cells were seeded and cultured for 24 h. Then, the cells were subjected to hypoxia exposure. The level of mitochondrial membrane potential (MMP) was analyzed with a mitochondrial membrane potential assay kit with JC-1 (Beyotime, Shanghai, China) according to the manufacturer’s instructions.

### 4.4. Apoptosis Detection

The cells were seeded and cultured for 24 h. Then, the cells were subjected to hypoxia exposure. The level of apoptosis was analyzed with an Annexin V-FITC apoptosis detection kit (Beyotime, Shanghai, China) according to the manufacturer’s instructions.

### 4.5. Whole-Transcriptome Sequencing Assay

Whole-transcriptome sequencing assays were performed by Lianchuan Biotechnology (No. 758, Weiken Street, Qiantang District, Hangzhou, China). The total RNA was isolated and purified via the TRIzol reagent (Invitrogen, Carlsbad, CA, USA) following the manufacturer’s instructions. The RNA quantity was measured by a NanoDrop ND-1000 (NanoDrop, Wilmington, DE, USA) spectrophotometer, and the RNA integrity was assessed with an 2100 Bioanalyzer (Agilent, Santa Clara, CA, USA).

The total RNA was depleted of ribosomal RNA according to the manufacturer’s instructions for the Ribo-Zero™ rRNA Removal Kit (Illumina, San Diego, USA). Sequencing (PE150) was performed on an HiSeq 6000 (Illumina, San Diego, CA, USA) following the manufacturer’s protocol. Cutadapt (version 1.10) was used to clean the reads. HISAT2 was used to map reads to the genome of each species. CIRCExplorer2 (version 2.2.6) and CIRI (version 2.0.2) were used to identify circRNAs. The mapped reads from each sample were assembled via StringTie (version 1.3.0). Then, all transcripts from the samples were merged using Perl scripts to reconstruct a comprehensive transcriptome. When identifying lncRNAs, transcripts that overlapped with known mRNAs and transcripts shorter than 200 bp were discarded. CPC and CNCI were subsequently used to predict transcripts with coding potential. The remaining transcripts were considered to be lncRNAs. StringTie (version 1.3.0) and edgeR (version 3.22.5) were used to estimate the expression levels of each transcript and to perform differential analysis. The differentially expressed mRNAs, lncRNAs, and circRNAs with FC ≥ 2 and *p* < 0.05 were selected.

The total RNA was converted to a small RNA sequencing library using a TruSeq Small RNA Sample Prep Kit according to the manufacturer’s procedure (Illumina, San Diego, USA). The HiSeq 2500 platform(Illumina, San Diego, CA, USA) was used for sequencing (SE50). The raw reads were subjected to ACGT101-miR (version 4.2, LC Sciences, Houston, TX, USA). Adapter dimers, junk, low complexity sequences, other RNAs, and repeats were removed. Subsequently, unique sequences with lengths of 18~26 nucleotides were mapped to species-specific precursors in miRBase 22.0 by a BLAST search (accessed on 25 May 2023) to identify miRNAs. The differentially expressed miRNAs with FC ≥ 1.5 and *p* < 0.05 were selected. TargetScan (version 5.0) and Miranda (version 3.3a) were used to identify miRNA binding sites.

### 4.6. Small RNA Microarray

Small RNA microarray assays were performed by Shu Pu (2168 Chenhang Highway, Building 10C, 4th Floor, Pujiang Smart Plaza, Shanghai, China) Biotechnologies LLC. The total RNA was isolated and purified via the TRIzol reagent (Invitrogen, Carlsbad, CA, USA) following the manufacturer’s instructions. The RNA quantity was measured with a NanoDrop ND-1000 (NanoDrop, Wilmington, DE, USA) spectrophotometer, and the RNA integrity was assessed with an Agilent 2100 Bioanalyzer. For small RNA microarray profiling, 100 ng of the total RNA was first dephosphorylated with 3 units of T4 polynucleotide kinase (T4 PNK) at 37 °C for 40 min to remove both (P) and (cP) chemical groups from the 3′ end of RNAs to form a 3-OH end. The reaction was terminated at 70 °C for 5 min and cooled immediately to 0 °C. An amount of 7 uL of DMSO was added and heated to 100 °C for 3 min to denature the RNAs and was chilled immediately to 0 °C. RNA end labeling was performed by adding ligase buffer, BSA, final 50 mM pCp-Cy3, and 15 units of T4 RNA ligase in a 28 uL reaction at 16 °C overnight. The labeled sample mixture was hybridized to the microarray (Arraystar Mouse small RNA Microarray, 8 × 15 K, Rockville, MD, USA) at 55 °C for 20 h. The slides were scanned on an G2505C microarray scanner (Agilent, Santa Clara, CA, USA). Agilent Feature Extraction software (version 11.0.1.1) was used to analyze the acquired array images. Differentially expressed small RNAs with FC ≥ 1.5 and *p* < 0.05 were selected.

### 4.7. Quantitative Reverse Transcription PCR (RT-qPCR)

According to the manufacturer’s instructions, the total RNA was extracted from bEnd.3 cells with the TRIzol reagent (Invitrogen, Carlsbad, CA, USA), and miRNAs were isolated with a SanPrep Column microRNA Extraction Kit (Sangon Biotech, Shanghai, China). For mRNAs, lncRNAs, and circRNAs, the 5× All-In-One RT MasterMix (Abm, Richmond, BC, Canada) was used for reverse transcription. The cDNA and primers were mixed with 2× M5 HiPer SYBR Premix EsTaq (Mei5bio, Beijing, China) and then subjected to RT–qPCR. The levels of miRNAs were confirmed via miRNA First Strand cDNA Synthesis Kits and microRNA qPCR Kits (SYBR Green Method) (Sangon Biotech, Shanghai, China) according to the manufacturer’s instructions. The levels of the tsRNAs were confirmed via Shu Pu (Shanghai, China) Biotechnologies LLC. The 2−∆∆Ct method was used to calculate the mRNA and miRNA expression levels. The sequence of primers is in the Supplementary File.

### 4.8. TMT-Based Proteomic Quantification

TMT quantitative proteomic assays were provided by Lianchuan Biotechnology (No. 758, Weiken Street, Qiantang District, Hangzhou, China). The cells were lysed in 8 M urea/50 mM Tris-HCl containing a protease inhibitor cocktail to extract proteins. Trypsin was used for protein digestion, and the TMT reagent was used to label the sample. An Orbitrap Exploris™ 480 mass spectrometer (Thermo Fisher Scientific, San Jose, CA, USA) was used for DDA (data-dependent acquisition) mode detection. The MaxQuant (version 2.1.4.0) software was used to analyze the TMT-plexed MS/MS raw data. Differentially expressed proteins were defined as those with FC ≥ 1.2 and *p* < 0.05.

### 4.9. Untargeted Metabolomics

Untargeted metabolomic assays were provided by Lianchuan Biotechnology (No. 758, Weiken Street, Qiantang District, Hangzhou, China). The collected samples were thawed on ice, and metabolites were extracted with 80% methanol buffer. A Q Exactive high-resolution tandem mass spectrometer (Thermo Fisher Scientific, San Jose, CA, USA) was used to detect the metabolites eluted from the column. The MS data was preprocessed using the XCMS software (version 3.5.1). LC−MS raw data files were converted into the mzXML format and then processed by the XCMS, CAMERA, and metaX toolboxes in R software (version 4.0). Each ion was identified on the basis of the retention time (RT) and *m*/*z* data. An in-house metabolite fragment spectrum library was used to validate the metabolite identification. The online KEGG and HMDB databases were used to annotate the metabolites by matching the exact molecular mass data (*m*/*z*) of the samples with those from the database. The differentially expressed metabolites with FC ≥ 1.5, *p* < 0.05, and VIP ≥ 1 were selected.

### 4.10. Visualization of Results

OmicShare https://www.omicshare.com/tools (accessed on 25 May 2023) was used for PCA, differential gene enrichment analysis, and the visualization of enrichment circles and nine-quadrant plots. The R package “corrplot” was used for Pearson correlation analysis and visualization. The R package “clusterProfiler” was used for enrichment analysis and GSEA. The R package “ggplot2” was used for volcano plots, scatter volcano plots, GSEA plots, and bubble plots. The R packages “GseaVis” and “enrichplot” were used for GSEA visualization. The R package “GSVA” was used for GSVA, the “limma” package was used for differential analysis, and the “pheatmap” package was used to generate heatmaps. The R packages “ggsankey” and “ggplot2” were used to generate Sankey plots. The STRING database https://string-db.org/ (accessed on 18 November 2023) was used to construct the PPI networks. Cytoscape (version 3.9.1) was used for protein–protein interaction (PPI) network visualization and analysis. CytoHubba in Cytoscape was used to screen the hub genes in the PPI networks. The MCODE plugin in Cytoscape was used to filter highly interconnected subnetworks. MetaboAnalyst 6.0 https://www.metaboanalyst.ca/ (accessed on 12 March 2024) was used for combined transcriptome and metabolomic KEGG analysis.

### 4.11. Statistical Analysis

GraphPad Prism version 8.3.0 was used to analyze the data. The results are expressed as the mean ± SEM from at least three independent experiments. Differences between groups were analyzed by Student’s *t*-test. *p* < 0.05 was considered to indicate statistical significance.

## 5. Conclusions

In conclusion, our findings suggest that hypoxia affected the ncRNA processing pathway and significantly changed the ncRNA levels such as miRNAs, tRNAs, tsRNAs, snoRNAs, lncRNAs, and circRNAs in BMECs. ncRNAs may play an important role in oxidative stress and cellular adaptation to hypoxia. Ultimately, we expect that our results will help facilitate an understanding of the pathological processes underlying BBB injury and provide potential targets for the treatment of BBB disorder-related cerebrovascular disease.

## Data Availability

The raw RNA and miRNA sequence data reported in this paper have been deposited in the Genome Sequence Archive (Genomics, Proteomics & Bioinformatics 2021) in the National Genomics Data Center (Nucleic Acids Res 2022), China National Center for Bioinformation/Beijing Institute of Genomics, Chinese Academy of Sciences (GSA: CRA022552 and CRA022523) and are publicly accessible at https://ngdc.cncb.ac.cn/gsa [GSA] [https://ngdc.cncb.ac.cn/gsa] [GSA: CRA022552/CRA022523].

## Supplementary Materials

The Supplementary Tables S1–S9 can be downloaded at: https://www.mdpi.com/article/10.3390/ijms26125629/s1.
