# Supplementary material for: Multiomics Analysis Reveals Role of ncRNA in Hypoxia of Mouse Brain Microvascular Endothelial Cells
Source: Int J Mol Sci. 2025 Jun 12;26(12):5629. doi: 10.3390/ijms26125629 (PMC12193265; doi:10.3390/ijms26125629)
Supplement: Supplementary file 1 [file ijms-26-05629-s001.zip › Supplementary Materials and Methods qRT-PCR primers.pdf]

Primer	5′-3′
U6-RT	AACGCTTCACGAATTTGCGT
U6-forward	CTCGCTTCGGCAGCACA
U6- reverse	AACGCTTCACGAATTTGCGT
mmu-miR-210-3p-RT	GTCGTATCCAGTGCAGGGTCCGAGGTATTCGC ACTGGATACGACTCAGCC
mmu-miR-210-3p-forward	TGTGCGTGTGACAGCGG
mmu-miR-210-3p-reverse	CGCAGGGTCCGAGGTATTC
mmu-miR-210-5p-RT	GTCGTATCCAGTGCAGGGTCCGAGGTATTCGC ACTGGATACGACCAGTGT
mmu-miR-210-5p-forward	CTACAAGCCACTGCCCACC
mmu-miR-210-5p-reverse	CGCAGGGTCCGAGGTATTC
mmu-miR-212-3p-RT	GTCGTATCCAGTGCAGGGTCCGAGGTATTCGC ACTGGATACGACTGGCCG
mmu-miR-212-3p-forward	GCCGCGTCTAACAGTCTCC
mmu-miR-212-3p-reverse	CGCAGGGTCCGAGGTATTC
mmu-miR-132-3p-RT	GTCGTATCCAGTGCAGGGTCCGAGGTATTCGC ACTGGATACGACCGACCA
mmu-miR-132-3p-forward	GCTCTACAGCCATGGTCGG
mmu-miR-132-3p-reverse	CGCAGGGTCCGAGGTATTC
mmu-miR-20a-5p-RT	GTCGTATCCAGTGCAGGGTCCGAGGTATTCGC ACTGGATACGACCTACCT
mmu-miR-20a-5p-forward	CCCCGCCTAAAGTGCTTATAG
mmu-miR-20a-5p-reverse	CGCAGGGTCCGAGGTATTC
mmu-miR-17-5p-RT	GTCGTATCCAGTGCAGGGTCCGAGGTATTCGC ACTGGATACGACCTACCT
mmu-miR-17-5p-forward	CCGCCAAAGTGCTTACAGTG
mmu-miR-17-5p-reverse	CGCAGGGTCCGAGGTATTC
Actb-forward	CCGTAAAGACCTCTATGCCAAC
Actb - reverse	AGGAGCCAGAGCAGTAATCT
mmu_circ_0000037-forward	GCAACTTTATCATCAGCAGCC
mmu_circ_0000037-reverse	TACAACCCTCTTTGGGGTC
circRNA958-forward	AGTAGGTGCTAGTTCCGCAG
circRNA958-reverse	TCAAGTTTACCCAAAGAGAGG
ENSMUST00000173672-forward	CAGGTGGACTCACTCATC
ENSMUST00000173672-reverse	AACAGGTCTCAGAACTAAGG
ENSMUST00000152754-forward	GTTCCTTCCTTGCTTGAGAG
ENSMUST00000152754-reverse	GCCAAAGAGGTTTACACACT
ENSMUST00000134244-forward	TGATGAATGCTATGGGAATGG
ENSMUST00000134244-reverse	GCACCGCCTTAACCTTAG
